# Presence and Immunoreactivity of *Aggregatibacter actinomycetemcomitans* in Rheumatoid Arthritis

**DOI:** 10.3390/pathogens13050368

**Published:** 2024-04-29

**Authors:** Anna Svärd, Riccardo LoMartire, Klara Martinsson, Carina Öhman, Alf Kastbom, Anders Johansson

**Affiliations:** 1 Center for Clinical Research Dalarna, Uppsala University, 791 82 Falun, Sweden; anna.svard@regiondalarna.se (A.S.); riccardo.lomartire@regiondalarna.se (R.L.); 2 Department of Rheumatology, Linköping University Hospital, 581 85 Linköping, Sweden; alf.kastbom@liu.se; 3 School of Health and Welfare, Dalarna University, 791 88 Falun, Sweden; 4 Division of Inflammation and Infection, Department of Biomedical and Clinical Sciences, Linköping University, 581 83 Linköping, Sweden; klara.martinsson@liu.se; 5 Department of Odontology, Umeå University, 901 87 Umeå, Sweden; carina.ohman@umu.se

**Keywords:** *Aggregatibacter actinomycetemcomitans*, salivary concentrations, rheumatoid arthritis, systemic biomarker

## Abstract

The presence of periodontal pathogens is associated with an increased prevalence of rheumatoid arthritis (RA). The systemic antibody response to epitopes of these bacteria is often used as a proxy to study correlations between bacteria and RA. The primary aim of the present study is to examine the correlation between the presence of *Aggregatibacter actinomycetemcomitans* (Aa) in the oral cavity and serum antibodies against the leukotoxin (LtxA) produced by this bacterium. The salivary presence of Aa was analyzed with quantitative PCR and serum LtxA ab in a cell culture-based neutralization assay. The analyses were performed on samples from a well-characterized RA cohort (*n* = 189) and a reference population of blood donors (*n* = 101). Salivary Aa was present in 15% of the RA patients and 6% of the blood donors. LtxA ab were detected in 19% of RA-sera and in 16% of sera from blood donors. The correlation between salivary Aa and serum LtxA ab was surprisingly low (rho = 0.55 [95% CI: 0.40, 0.68]). The presence of salivary Aa showed no significant association with any of the RA-associated parameters documented in the cohort. A limitation of the present study is the relatively low number of individuals with detectable concentrations of Aa in saliva. Moreover, in the comparison of detectable Aa prevalence between RA patients and blood donors, we assumed that the two groups were equivalent in other Aa prognostic factors. These limitations must be taken into consideration when the result from the study is interpreted. We conclude that a systemic immune response to Aa LtxA does not fully reflect the prevalence of Aa in saliva. In addition, the association between RA-associated parameters and the presence of Aa was negligible in the present RA cohort.

## 1. Introduction

Rheumatoid arthritis (RA) is an inflammatory disease that can display autoantibody production and systemic disease manifestations [1,2]. Autoantibodies to citrullinated proteins (ACPAs) are specific markers of RA and are usually assayed as antibodies to citrullinated peptides (anti-CCP) [3]. For many years, RA has been suggested to be clinically and pathologically associated with periodontitis [4]. Periodontitis is a bacteria-induced inflammatory disease that degrades the tooth-supporting structures, alveolar bone and connective tissue [5]. Interestingly, Konig and co-workers [6] showed that the citrullinome in periodontitis sometimes reflected patterns of hypercitrullination observed in the rheumatoid joint. Among the periodontal pathogens, *Aggregatibacter actinomyctemecomitans* (Aa), but not other bacterial species, induced hypercitrullination in host neutrophils [6]. It was shown that the molecular mechanism by which Aa triggers the dysregulated activation of citrullinating enzymes in neutrophils mimics autoantigen citrullination in the RA joint. Aa is a Gram-negative facultative anaerobic bacterial species that is associated with aggressive forms of periodontitis [7,8]. 

Due to the high genetic diversity of Aa, the bacterium exists as a harmless commensal and as an exogene pathogen [9]. A highly virulent genotype of the bacterium is the JP2, which has an enhanced production of a leukotoxin (LtxA) [10]. Detection of the JP2 genotype of Aa was initially restricted to some regions of Africa, while it today can be sporadically found in patients of many different geographic origins around the world [11]. Adolescents carrying the JP2 genotype of Aa are at significantly enhanced risk to develop periodontitis [12]. LtxA produced by this bacterium is closely linked to the initiation of degenerative processes involved in many diseases, like periodontitis and rheumatoid arthritis [13,14]. The toxin is a large pore-forming protein that specifically binds to the β_2_ integrin LFA-1 (CD11a/CD18) expressed by human leukocytes [15]. The interaction of LtxA with human neutrophiles induces an extracellular release of trap-like structures that contain citrullinated proteins [6,16]. The NLRP3 inflammasome is activated by LtxA and is important in the pathogenesis of both RA and periodontitis [17,18]. These properties of the periodontal pathogen Aa and its LtxA indicate a potential relationship between RA and the prevalence of Aa [19]. It has been reported that an RA patient with periodontitis colonized with the highly leukotoxic JP2 genotype of Aa one year after the eradication of Aa remained free of arthritis and anti-CCP antibodies [20]. A commonly used strategy for the determination of Aa prevalence on an individual basis is an analysis of systemic antibodies against surface epitopes unique for this bacterium [21]. Systemic antibodies with reactivity against LtxA have shown to be significantly enhanced in RA patients compared with healthy controls when examined in a US population [6]. Contrary to this observation, systemic LtxA antibodies, when analyzed in a Swedish cohort, were not significantly associated to RA [22]. Moreover, one previous study on a Japanese population showed that systemic IgG responses to Aa in patients with RA were significantly lower than those in controls [23].

In addition to the immunodetection of Aa-LtxA antibodies, the systemic capacity to neutralize the activity of LtxA has been analyzed [24,25,26]. The presence of systemic LtxA-neutralizing capacity has been shown to correlate significantly with the occurrence of LtxA antibodies, while the association with periodontitis has not been established [27,28,29]. We have previously examined the prevalence of systemic LtxA neutralization in relation to RA and associated systemic risk markers without any conclusive result for the associations between LtxA neutralization and RA development [30]. Aa has been shown to produce virulence factors with the capacity to affect a proper host response against this bacterium [31,32]. However, it is not yet known how the presence of systemic LtxA antibodies correlates with the amount of Aa in the oral cavity. RA has been associated with an impaired host response in vaccine studies [33,34]. Taken together, these findings indicate the importance of investigating whether systemic antibodies against Aa and its LtxA reflect the prevalence of the bacterium in the oral cavity in RA patients.

The primary aim of the present study is to examine the correlation between the amount of Aa in the oral cavity and the level of systemic Aa-LtxA antibodies in serum. A secondary aim is to examine the salivary presence of Aa in relation to the occurrence of RA-associated serological and clinical parameters.

## 2. Materials and Methods

### 2.1. Study Populations and Samples

The study populations have previously been described in detail [35]. Briefly, 196 individuals with established RA from the County of Dalarna, Sweden, were included in the cross-sectional “Secretory antibodies in Rheumatoid Arthritis” (SARA) study with enrolment 2012–2013. RA patients were randomly selected among planned follow-up visits at the Rheumatology Clinic, Falun Hospital, Sweden. Healthy blood donors (*n* = 101) were recruited from the local blood donor center and referred to the Rheumatology Clinic for blood and saliva sampling. Demographic and clinical parameters of the two study cohorts are summarized in Table 1. Participants were required to provide at least 0.5 mL of saliva during a 10-min sampling time; otherwise, they were excluded from the study. Paired saliva and serum samples were collected at the same visit at the Rheumatology Clinic. Participants were asked to restrain from eating, drinking other liquids than water, brushing teeth, or smoking 1 h before saliva sampling. Passive secretion was used for saliva sampling, i.e., the study participant leaned forward and drooled for 10 min into a test tube placed on ice. After the disruption of mucus fibers by pipetting a few times, the saliva samples were centrifuged for 5 min at 5000× *g*. Serum samples were also centrifuged for 5 min at 5000× *g*. Subsequently, serum and saliva samples were stored at −80 °C until further analyses.

RA patients’ disease activity was registered on the day of sampling and measured by the Disease Activity Score of 28 joints (DAS28) using erythrocyte sedimentation rate (ESR).

Information on smoking and oral health was obtained using a self-report questionnaire provided by the Epidemiological Investigations in RA (EIRA) Study [36].

### 2.2. Analysis of Total IgA in Saliva

The total IgA in saliva was quantified using commercially available immune assays (IBL International, Hamburg, Germany).

### 2.3. Autoantibody Analyses

IgG ACPAs in serum were analyzed previously, using the second-generation anti-CCP immunoassay (Svar Life Science, Malmö, Sweden). IgA ACPAs in serum and saliva were also analyzed previously in a similar way but using an anti-human α-chain antibody as secondary antibody [37].

Serum secretory component-containing (SC) ACPAs were measured by modifying commercially available anti-CCP ELISA kits (Euro-Diagnostica, Malmö, Sweden) as described elsewhere [38]. Briefly, SC ACPA were analyzed by diluting serum samples 1:25, and the detection antibody was diluted 1:2000 (polyclonal goat antibody conjugated to horseradish peroxidase, GAHu/SC/PO, Nordic Biosite, Sweden). Incubation and washing were performed according to instructions by the manufacturer. A 7-step standard curve was calculated by diluting a serum sample high in SC ACPAs.

### 2.4. Leukotoxin (LtxA) Antibody Assay

Anti-LtxA antibodies in serum were analyzed for their LtxA neutralizing capacity, which was detected as a reduction in cell damage and subsequent inhibited leakage of neutral red upon exposure to purified LtxA [39]. THP-1 cells in RPMI-10% fetal bovine serum (FBS)-50 nM phorbol myristate acetate were seeded at 1 × 10^6^ cells/mL in flat 96-well plates and incubated at 37 °C 5% CO_2_ overnight. The cells were washed with RPMI, patient serum and LtxA added in triplicates and incubated for 2 h at 37 °C 5% CO_2_. The medium was removed, and 0.04 mg/mL neutral red diluted in RPMI-10% FBS was added, incubated 90 min at 37 °C 5% CO_2_ and washed with PBS pH 7.4. Then, 50% EtOH with 1% acetic acid was added to lyse the cells. After 10 min of incubation, the optical density (OD) was read at 650 nm (TECAN Sunrise, CA, USA). The anti-LtxA antibody neutralization capacity in percent was calculated by dividing the serum sample OD with the maximum cell viability OD (incubation with FBS only) × 100. Serum samples inhibiting LtxA cell lysis ≥30% were classified as positive and <30% were classified as negative regarding anti-LtxA presence [27].

### 2.5. qPCR-Based Quantification of Salivary A. actinomycetemcomitans

This method has been previously described in detail [40]. Briefly, stimulated saliva was collected and the Viral DNA extraction kit (DiaSorin AB, Dublin, Ireland) was used for the DNA isolation, and for the procedure, an automated extraction instrument was used (Liaison^®^ IXT, Diasorin AB, Ireland). DNA was extracted from 550 μL of the sample mixture and eluted in a volume of 100 μL. Standard suspensions of the Aa (JP2 genotype HK1651) (10^8^–10^1^ cells/mL), prepared in PBS buffer, were treated as described above. The samples and the standard solutions were stored at +4 °C until use. Quantification of the total concentration of *A. actinomycetemcomitans* in the DNA samples was performed according to Claesson et al., 2019 (PMID: 30847232). Briefly, the PCR mixture (10 μL) contained 5 μL Kapa Syber Green (KK 4601; Kapa Biosystems, Boston USA), 4 μL template, and 1 μL of a primer mix specific for the LtxA (0.5 μM/primer, F: CTAGGTATTGCGAAACAATTT, R: CCTGAAATTAAGCTGGTAATC). The PCR program was as follows: hold/time 95°/10 m, cycling/time 95°/10 s, cycling/time 55°/5 s and 45 cycles.

### 2.6. Ethics

The ethics review board in Uppsala, Sweden, approved of the study, and all participants signed written informed consent (Uppsala: 2011/159).

### 2.7. Statistical Analyzes

The prevalence point estimate (Wilson’s 95% confidence interval) of both detectable salivary Aa ≥ 100 bacteria/mL and detectable serum LtxA ab ≥ 30% was computed for the RA cohort and blood donors separately (DescTools v0.99.48 in R v4.2.3). To test the equality of the prevalence between the cohorts, we used a z-test of two independent proportions (R v4.2.3). Next, the monotonic correlation between salivary Aa and serum LtxA ab was quantified using Spearman’s rho on left-censored data at the detection limit [41] with confidence intervals based on the bias-corrected and adjusted bootstrap method with 10,000 replications (boot v1.3-28.1 in R v4.2.3) [42]. In the final phase, we restricted the analyses to the RA patients. Within this subset, we first quantified the correlation between detectable salivary Aa and RA-associated risk markers using Spearman’s rho; then, we estimated the association between detectable salivary Aa and detectable ACPA using Pearson’s chi-square test and finally compared both DAS28 and total IgA in saliva between patients with and without detectable salivary Aa using the Mann–Whitney U test. All computations were based on complete cases.

## 3. Results

### 3.1. Salivary A. actinomycetemcomitans and Serum LtxA Antibodies in Both Cohorts

Salivary Aa ≥ 100 bacteria/mL was found in 14.8% (95% CI: 10.5%, 20.6%) of RA patients and in 5.9% (95% CI: 2.8%, 12.4%) of blood donors. Meanwhile, LtxA-neutralizing antibodies ≥ 30% inhibition in serum were found in 18.8% of RA patients (95% CI: 13.9%, 25.0%) and 16.0% (95% CI: 10.1%, 24.4%) of blood donors (Table 2).

### 3.2. Association between Salivary Aa and Systemic LtxA ab in Both Cohorts

**Figure 1:**
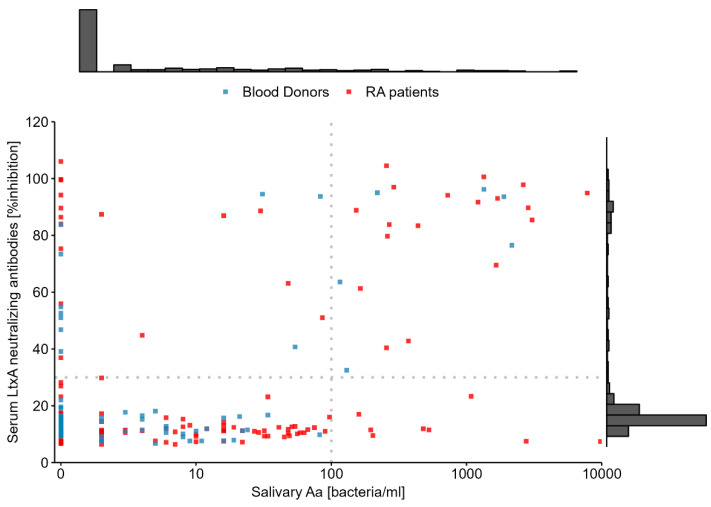
Relationship between salivary Aa and systemic LtxA ab in patients with RA (red points) and healthy blood donors (blue points). Salivary Aa is presented on the natural log scale with a constant of one added to zero values before transformation. The dotted lines mark the detection limit for each variable, and each variable´s distribution is illustrated by the marginal histograms.

Based on data from 279 individuals, we observed a moderately strong positive correlation of 0.55 (95% CI: 0.40, 0.68) between salivary Aa and LtxA-neutralizing antibodies, which is illustrated in Figure 1. Correlation coefficients were similar when estimated separately for RA patients (rho = 0.54; 95% CI: 0.36, 0.69) and blood donors (rho = 0.60; 95% CI: 0.38, 0.80).

Among the 34 individuals that tested positive for Aa in saliva, 24 also tested positive for LtxA-neutralizing antibodies in serum, while eight tested negative and two had no recorded data. Interestingly, the eight incongruent individuals, with Aa in saliva but no LtxA antibody in serum, were all RA patients. Moreover, they were females 49–66 years old and 6/8 were smokers. The median (interquartile range) of total IgA in saliva for these eight individuals was 90 µg/mL (106) compared to 55 µg/mL (50) for the 24 individuals that were positive for both saliva Aa and serum LtxA ab.

### 3.3. Association between Aa and ACPA, Disease Activity and Treatment in RA Patients

Among RA patients, 80% were positive for IgG ACPA in serum, 45% for IgA ACPA in serum and 12% for IgA ACPA in saliva. When comparing ACPA status between RA patients with and without Aa in saliva, no differences of importance were observed (Table 3). Also, DAS28 was similar among RA patients with Aa in saliva (median: 2.8; IQR: 1.9) and RA patients without Aa in saliva (median: 3.2; IQR: 1.3; P = 0.27).

**Figure 2:**
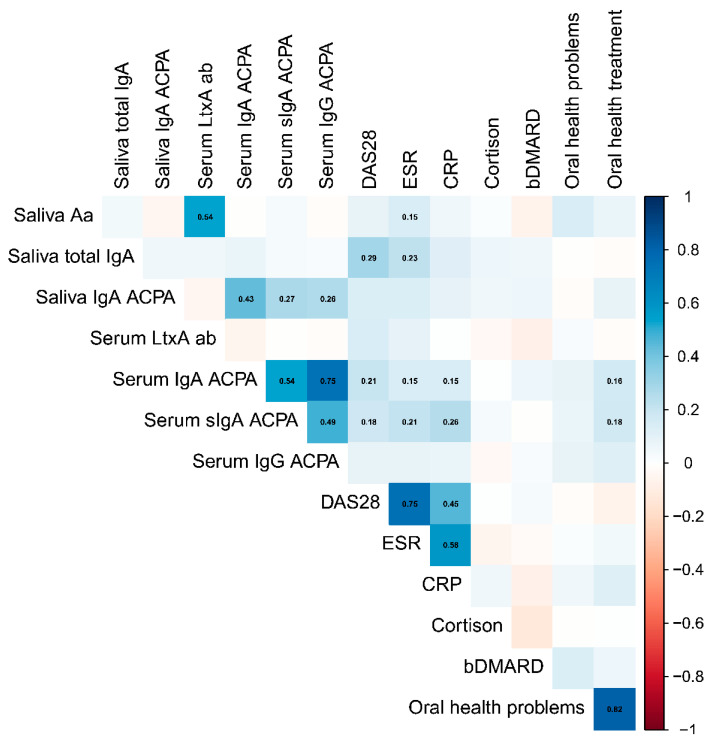
Plot of monotonic correlations between Aa markers and RA-associated parameters based on data left censored at the detection level. Blue color and red color indicate positive and negative correlations, respectively. Point estimates shown for correlations significant at 0.05 level only. The different parameters are described in material and methods (Section 2.1,Section 2.2 and Section 2.3).

Correlations between detectable salivary Aa and ESR, CRP, oral health and RA treatment were also investigated among the RA patients, as illustrated in Figure 2. No correlations of clinical importance were observed for salivary Aa or LtxA-neutralizing antibodies, besides the moderately strong positive correlation between the two as mentioned above.

## 4. Discussion

The association between RA and periodontitis is well established, while the role of the periodontal pathogens still is unclear [19,43]. In the present study, we examined the prevalence of Aa in saliva samples from two different cohorts: one consisted of patients diagnosed with RA and one consisted of healthy blood donors [35]. The prevalence of salivary Aa was 15% in the RA cohort and 6% among the blood donors. Aa LtxA-neutralizing ab in serum was detected in 19% of the RA patients and in 16% of the blood donors. The prevalence of salivary Aa and serum LtxA ab corresponds to levels previously found in periodontally healthy individuals rather than in periodontitis patients [44,45]. The correlation between saliva Aa and serum LtxA ab in the present study was surprisingly low, indicating that systemic immunoreactivity against Aa epitopes does not fully reflect the presence of this bacterium in the oral cavity. This indicates that collected biobank samples of plasma or serum do not fully reflect the oral presence of the periodontal pathogens. Systemic antibodies to Aa or LtxA reflect previous immunoreactivity against the bacterium, while detection of the bacterium in the oral cavity shows the presence at the time for sampling [21,29]. These circumstances have to be considered in the interpretation of data from diseases like RA that involve treatment strategies with immunosuppressive effects [46].

Findings from the present study did not support a correlation between the prevalence of Aa and the levels of different RA-associated parameters documented in the RA patients (ACPAs, DAS28, ESR, CRP, self-reported oral health, treatment with glucocorticoids or bDMARDs). A limitation in the present study is the lack of periodontal registrations. In addition, our results need to be interpreted in light of the low prevalence of detectable salivary Aa. However, the low prevalence of Aa and self-reported oral health problems in the present cohort indicates a generally good periodontal status among the study participants. A recent case-control study in a Chinese population showed that the presence of Aa was more devasting for RA patients with periodontitis compared to periodontally healthy RA patients [47]. This indicates that differences in the periodontal conditions between various studies might contribute to the diverging results on the correlation between RA-associated parameters and presence of periodontal pathogens [6,22]. Periodontitis-associated bacteria can be detected in the oral cavity of both periodontally healthy individuals and in periodontitis patients [5]. In periodontitis, the epithelial barrier function is impaired, which promotes the translocation of periodontal bacteria to the peripheral circulation [48]. The distribution of periodontal microbes into the peripheral circulation is associated with extra-oral inflammation and with several systemic diseases [49]. DNA of periodontal pathogens has been detected in synovial fluid, which indicated that these bacteria may play a role in the pathogenesis of RA [50,51].

There is a need for a suitable alternative to complete clinical periodontal examinations, which are both time consuming and expensive [52]. Radiographic periodontal bone loss shows a strong correlation to periodontitis and might be a suitable tool to simplify periodontal registrations in future population studies [44]. This technique has been successfully used to determine the correlation between periodontitis and RA; however, this study lacks data regarding the presence of periodontal microbes [53]. Aa produces molecules with virulence mechanisms that are closely linked to the pathogenesis of RA [6]. The association of Aa with RA is focused on the production of a leukotoxin (LtxA) with the capacity to induce dysbiosis in the host response [51]. The LtxA-induced virulence mechanisms involve the capacity to promote the citrullination of host proteins [54]. Aa can be found in the oral cavity of both periodontally healthy and periodontally diseased individuals [5,55].

Interestingly, in comparative analyses between the prevalence of Aa in saliva and serum LtxA, eight of the individuals from the two cohorts with Aa in saliva lacked serum LtxA ab. All these eight individuals were from the RA cohort, indicating a possible dysregulation of immune responses and subsequent impaired microbial host response in these RA patients. The association of RA with dysregulated immune response and autoimmunity may indicate impaired microbial host response [56]. In addition, Aa exhibit virulence properties that might contribute to avoid host response [31]. Among these properties, the bacterium can invade periodontal tissues and the ability to produce LtxA with the capacity to specifically activate and kill leukocytes [13,57]. The immunosuppressive effect of Aa has been reported by an enhanced systemic immune response to LtxA in a patient treated with antibiotics that eradicated the bacteria [20]. However, the observation of an impaired systemic immune response of Aa-colonized RA patients is based on few individuals and needs to be further confirmed in larger studies.

In conclusion, systemic immunoreactivity to the Aa bacterium does not completely reflect Aa in saliva. This is important knowledge when designing studies on Aa and possibly other periodontal pathogens. Also, we conclude that salivary Aa was not significantly associated with the levels of RA-associated parameters in serum from the RA cohort. However, these results need to be interpreted with care due to the low number of Aa-positive individuals.

## Figures and Tables

**Table 1:** Demographic and clinical parameters of the two study cohorts.

	RA Patients	Blood Donors
Number	196	101
Age ± SD	64(13)	49(14)
Men	40 (20%)	47 (47%)
Smoking	101 (52%)	36 (36%)
Oral health problems *	96 (50%)	23 (23%)
Oral treatment ^§^	53 (33%)	18 (18%)

* Self-reported experienced gum problem. ^§^ Having been subject to treatment of periodontal gum problem.

**Table 2:** Prevalence of salivary Aa and LtxA-neutralizing antibodies positive individuals in the two analyzed study cohorts.

	Saliva Aa		Serum LtxA ab	
	Total Analyzed (Missing)	Number Pos (%)	Total Analyzed (Missing)	Number Pos (%)
All	290 (7)	34 (11.7)	286 (11)	51 (17.8)
Blood donors	101 (0)	6 (5.9)	100 (1)	16 (16.0)
RA-cohort	189 (7)	28 (14.8)	186 (10)	35 (18.8)

**Table 3:** Levels of serum ACPA and saliva IgA stratified by detectable salivary Aa. No differences of importance in levels between the two groups were detected.

	RA Patients with Aa in Saliva	RA Patients without Aa in Saliva
Positive serum IgG ACPA	22 (79%)	128 (80%)
Positive serum IgA ACPA	12 (43%)	74 (46%)
Positive saliva IgA ACPA	2 (7%)	18 (11%)
Total IgA in saliva, median μg/mL (IQR)	61 (53)	62 (50)
SC ACPA in serum, median µg/mL (IQR)	52 (44)	68 (46)

## Data Availability

At Center for Clinical Research Dalarna, Uppsala University, Sweden.
